# Log file‐based patient‐specific quality assurance for MR‐guided radiation therapy with comprehensive motion management

**DOI:** 10.1002/acm2.70773

**Published:** 2026-08-25

**Authors:** Kai Yuan, Matthew Man‐hin Cheung, Louis Lee

**Affiliations:** ^1^ Medical Physics Division CUHK Medical Centre Hong Kong Hong Kong SAR China; ^2^ Department of Clinical Oncology, Faculty of Medicine The Chinese University of Hong Kong Hong Kong Hong Kong SAR China

**Keywords:** comprehensive motion management, log file, MR‐Linac, patient‐specific quality assurance, radiation therapy

## Abstract

**Background:**

Patient‐Specific Quality Assurance (PSQA) for Elekta Unity MR‐Linac treatments delivered under Comprehensive Motion Management (CMM) is challenging because beam gating, unexpected interruptions, and split sub‐segments introduce delivery complexities that are not fully addressed by conventional quality assurance workflows.

**Purpose:**

This study aimed to develop and evaluate a log file‐based PSQA program for Elekta Unity MR‐Linac treatments delivered under CMM.

**Methods:**

Log files were parsed to extract Multileaf Collimator (MLC) positions, jaw positions, and Monitor Units (MU) for each segment, with dedicated handling of CMM‐induced beam gating, unexpected beam interruptions, and split sub‐segments. Gantry‐resolved fluence maps reconstructed from the log files were compared with planned fluence maps using gamma analysis. The system was evaluated for 66 cases, including 3 completion plans and 2 baseline shift plans.

**Results:**

Among the 66 plans, 64 achieved passing gamma results, with a pooled average passing rate of 99.91 ± 0.21%.

**Conclusions:**

The proposed log file‐based PSQA framework demonstrates accuracy and clinical practicality for MR‐Linac treatments in CMM mode. It may serve as a reliable post‐treatment verification tool and reduce dependence on conventional phantom‐based QA.

## 1 INTRODUCTION

Elekta Unity (Elekta AB, Stockholm, Sweden) integrates a 1.5 T MR scanner, enabling online adaptive radiotherapy.^1^ The Unity system supports a maximum collimated field size of 57.4 cm × 22.0 cm in the IEC X and IEC Y directions at the isocentre plane, respectively. Lateral collimation is provided by X jaws traversing the cross‐plane direction, while longitudinal collimation is achieved via 160 Multileaf Collimators (MLC) operating in the in‐plane direction.

Unity's Motion Monitoring (MM) monitors the target without automatic gating and requires therapists to observe target motion and manually interrupt the beam when necessary. In contrast, Comprehensive Motion Management (CMM) provides real‐time target tracking using 2D cine Magnetic Resonance Imaging (MRI) and can automatically gate the beam when the target moves outside the planned region.^2^, ^3^ CMM supports four delivery strategies: a non‐respiratory mode, in which irradiation is paused only when target drift exceeds the tolerance, and three respiratory modes: free‐breathing (average), free‐breathing (exhale), and breath‐hold. In the free‑breathing modes, a continuously updated prediction model compensates for system latency to enable gating, whereas breath‑hold delivery uses no prediction and delivers radiation only after stable target positioning within tolerance is confirmed. In CMM, beam delivery is frequently gated, which can subdivide planned segments into multiple delivery sub‐segments. Several studies have comprehensively commissioned CMM using motion phantoms, evaluating beam latency and dosimetric performance and demonstrating good dose agreement as well as sub‐millimeter to low‐millimeter localization accuracy across various gating strategies and motion patterns.^2^, ^3^

If the tracking target shows a systematic drift outside the planned region on CMM images, a Baseline Shift (BLS) option can be used to compensate for the time‐averaged displacement. During a baseline shift, the mean target offset is sent to the online planning system so the plan's MLC apertures are shifted accordingly, while the MU and segment count remain unchanged. If treatment is interrupted due to unforeseen events (e.g., patient discomfort or a system interruption), a completion plan can be generated to deliver the remaining portion of the treatment. The completion plan includes only the undelivered beams and segments and can be adapted online using newly acquired images.

Unity's log files are recorded at 40 ms intervals and automatically capture key treatment delivery parameters, including MLC positions, jaw positions, Monitor Units (MU), and gantry angle.^4^, ^5^ The Rubicon optical leaf tracking system detects MLC positions through a fluorescence‐based mechanism calibrated during commissioning. Ultraviolet light excites ruby tips on the MLC leaves, producing infrared fluorescence that is captured by a dedicated infrared camera. This allows leaf positions to be monitored with real‐time precision.^4^ Log file‐based analysis is increasingly being adopted for Patient‐Specific Quality Assurance (PSQA) to provide detailed, time‐resolved insight into machine and delivery performance.^6^, ^7^, ^8^, ^9^, ^10^ A log file is generated each time the beam‐on button is pressed and is terminated when Automatic Field Sequencing (AFS) is completed, manually stopped by the radiation therapists, or interrupted by a system interruption. Under uninterrupted treatment delivery, the number of log files corresponds to the number of AFS sessions.

A previous study demonstrated the feasibility of using delivery log files for PSQA of Elekta Unity plans under conventional MM.^10^ With the transition from MM to CMM, the existing log‐file‐based PSQA workflow requires further refinement to account for CMM‐specific delivery characteristics. In addition, PSQA strategies for baseline shift and completion plans should be incorporated into the overall framework. This study aims to develop and evaluate log‐file‐based PSQA for Elekta Unity CMM deliveries, using gantry‐resolved fluence verification. Reconstructed log‐file fluence will be compared with Treatment Planning System (TPS) fluence at each gantry angle via gamma analysis.^10^

## 2 METHODS AND MATERIALS

### 2.1 Comparison of CMM to MM

In contrast to MM, beam delivery in CMM mode is frequently gated off under three conditions: degraded cine MR image quality (low quality factor), substantial deviation of target motion from the prediction model (low tracking accuracy), or the target moves outside the predefined positional or gating tolerances, triggering an Automatic Tolerance Check (ATC) failure.

**FIGURE 1:**
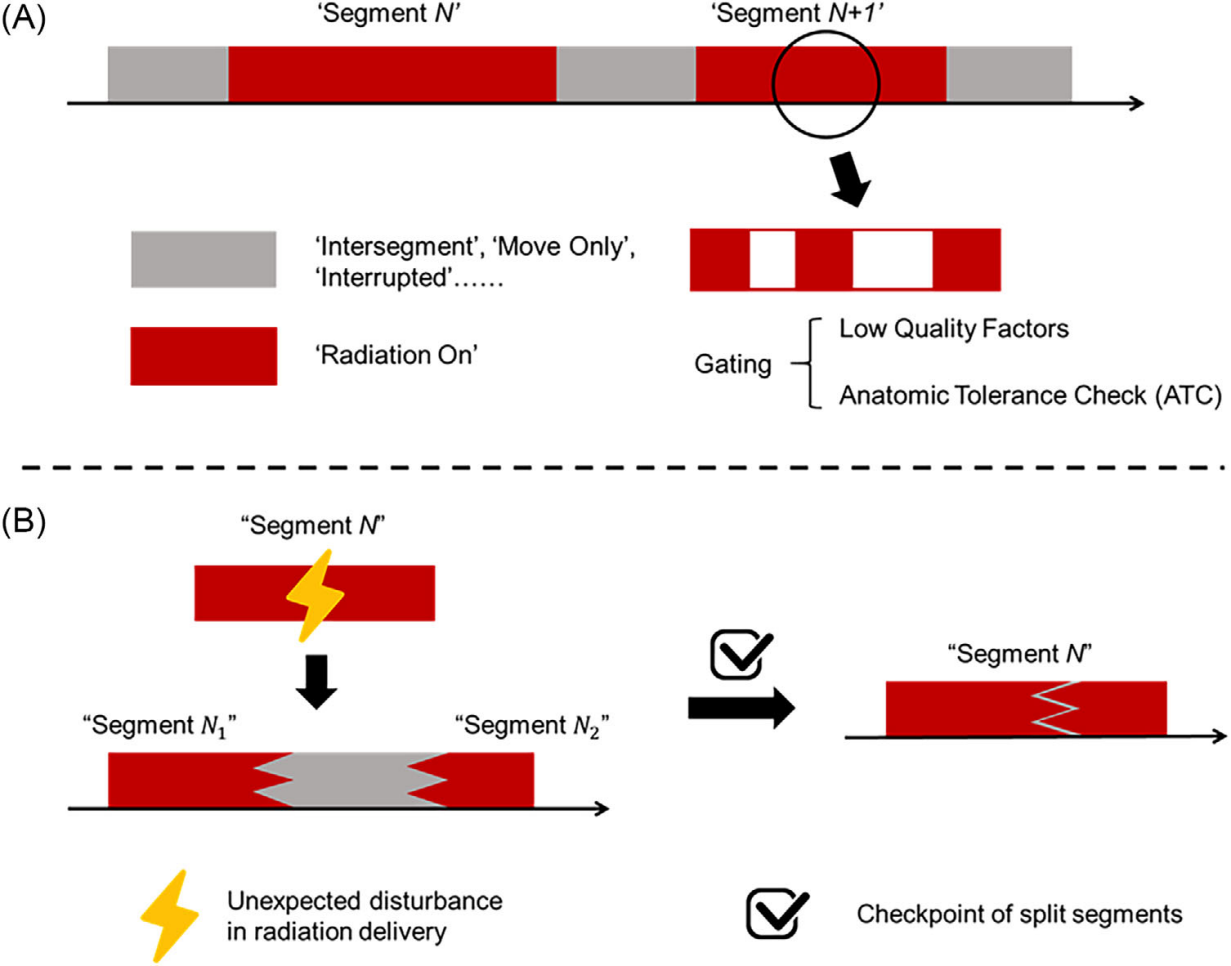
(a) The influence of CMM on the segments in the log files. White gaps indicate periods when the beam is gated off (due to low quality factor or ATC failure), during which the Linac State remains ‘Radiation On’ but no dose is delivered. (b) Checkpoint of split segments induced by unexpected disturbance in radiation delivery.

Individual segments were identified by detecting transitions into the “Radiation On” state, as recorded in the log file variable “Linac State.” The Elekta Unity system cycles through three distinct linac states during treatment delivery: “Intersegment,” “Move Only,” and “Radiation On.” Radiation is delivered exclusively during the “Radiation On” state. As illustrated in Figure 1(a), when the beam was gated off due to a low quality factor, low tracking accuracy, or an ATC failure, the linac state continued to be recorded as “Radiation On.” Whether the beam was gated off was recorded in the binary log file variable “Beam Hold Status”. The specific cause of gating could not be distinguished from the log file alone and had to be retrieved manually from the CMM Audit Log, which is not required for the automated PSQA calculation. By combining these state transitions with the step‐and‐shoot delivery characteristics of the Unity system, individual segments were reliably identified, enabling accurate assignment of the corresponding MLC positions, jaw positions, and MU to each segment. When the beam was gated off beyond a predefined machine time threshold, the Linac terminated the beam, recording ceased, and a new log file was generated upon the next beam‐on event. The same behavior applied to all other interruption types, including baseline shifts, unexpected interruptions, and manual beam‐off events.

The gating efficiency (*E*) was defined as:

$$E=\left(1-\frac{T_{H}}{T_{\mathrm{total}}}\right)\times 100\%$$

where $T_{H}$ denoted the fraction of time during which the beam was gated off due to low quality factors or failure to satisfy the ATC criteria, $T_{total}$ denoted the total beam‐on time.

### 2.2 Baseline shift plan

The BLS plan applied a minor adjustment to the daily adapted plan by sending the average target displacement to online Monaco for re‐adaptation, without requiring re‐imaging. Leaf positions were adjusted using the Segment Aperture Morphing (SAM) algorithm, while the original MUs, beam weights, and segment configuration were preserved.

Following a baseline shift, the TPS generated a complete plan with updated MLC positions encompassing all segments and fields, including those already delivered. The original plan and the BLS plan were therefore evaluated separately during PSQA. For the original plan, log files were used to identify the delivered fields and segments, and any undelivered portions were excluded from the comparison. For the BLS plan, the delivered segments and fields were similarly identified from the log files. The delivered portion of the BLS plan was then defined by matching segments from the end of the DICOM plan, while excluding segments that had already been delivered under the original plan.

### 2.3 Completion plan

When creating the completion plan, segments that had already been delivered were removed, and the revised plan was subsequently reviewed offline by a physicist. During PSQA, the original plan and the completion plan were evaluated separately. For the original plan, log files were used to identify which fields and segments had been delivered, and any undelivered portions were excluded from the comparison. For the completion plan, no such exclusion was necessary, and a direct comparison was made between the DICOM plan and the log files.

### 2.4 Unexpected beam‐off

The beam could be manually interrupted via the beam‐off button in response to an unexpected incident, or automatically halted by a machine interruption and subsequently resumed by pressing the reset button. In either case, the number of log files generated exceeded the number of planned AFS. Furthermore, when a beam interruption and restart occurred within a single segment, two consecutive segments in the log file shared identical MLC positions, provided that segments were identified solely on the basis of linac state transitions. As illustrated in Figure 1(b), a checkpoint mechanism was therefore introduced to determine whether two consecutive segments with identical MLC positions represented two distinct planned segments or constituted split sub‐segments of the same original segment.

### 2.5 Data analysis

**FIGURE 2:**
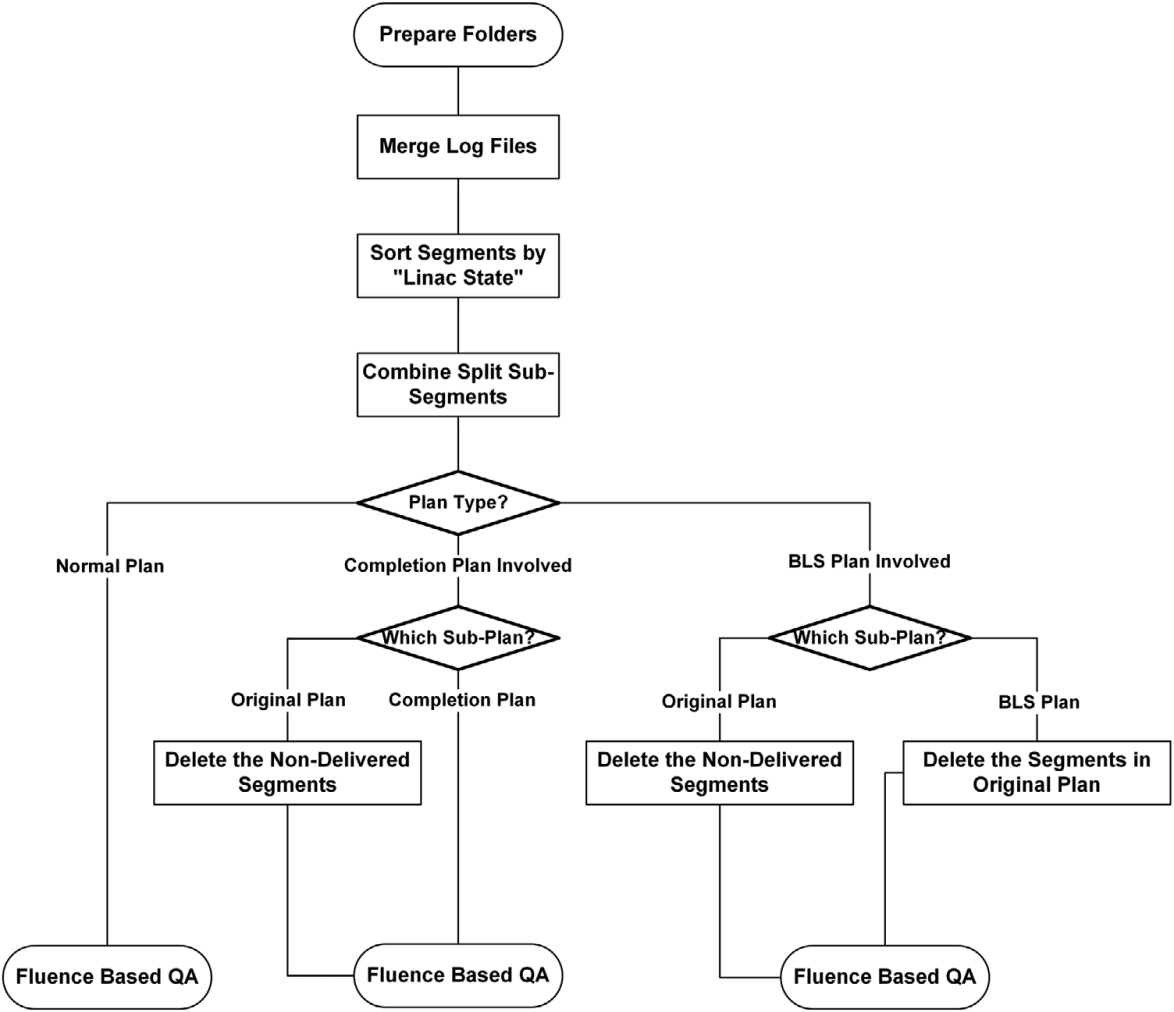
The flowchart illustrating the handling of CMM‐induced beam gating events, unexpected beam interruptions, and split sub‐segments in normal plans, completion plans and baseline shift plans. Split sub‐segments are combined specifically when an unexpected beam interruption splits a single planned segment into two consecutive segments with identical MLC positions.

The handling of CMM‐induced beam gating events, unexpected beam interruptions, and split sub‐segments across normal plans, completion plans, and baseline shift plans is illustrated in Figure 2. For both the TPS plan and the log files, a fluence map at the isocentre plane was reconstructed for each gantry angle by accumulating the fluence contributions of individual beam segments, each weighted by its corresponding MU value. Fluence maps derived from the TPS plan and the log files were compared using gamma analysis at a resolution of 0.1 mm. Consistent with the previous study, the distance‐to‐agreement and dose difference criteria were set to 1 mm and 1%, respectively. A threshold of 10% of the global maximum fluence was applied to restrict the analysis to clinically relevant regions.^10^ A gamma index in the range of 0–1 indicated a passing point, and a field was considered to meet the acceptance criterion if more than 98% of evaluated points achieved a passing gamma index.

### 2.6 User interface design

**FIGURE 3:**
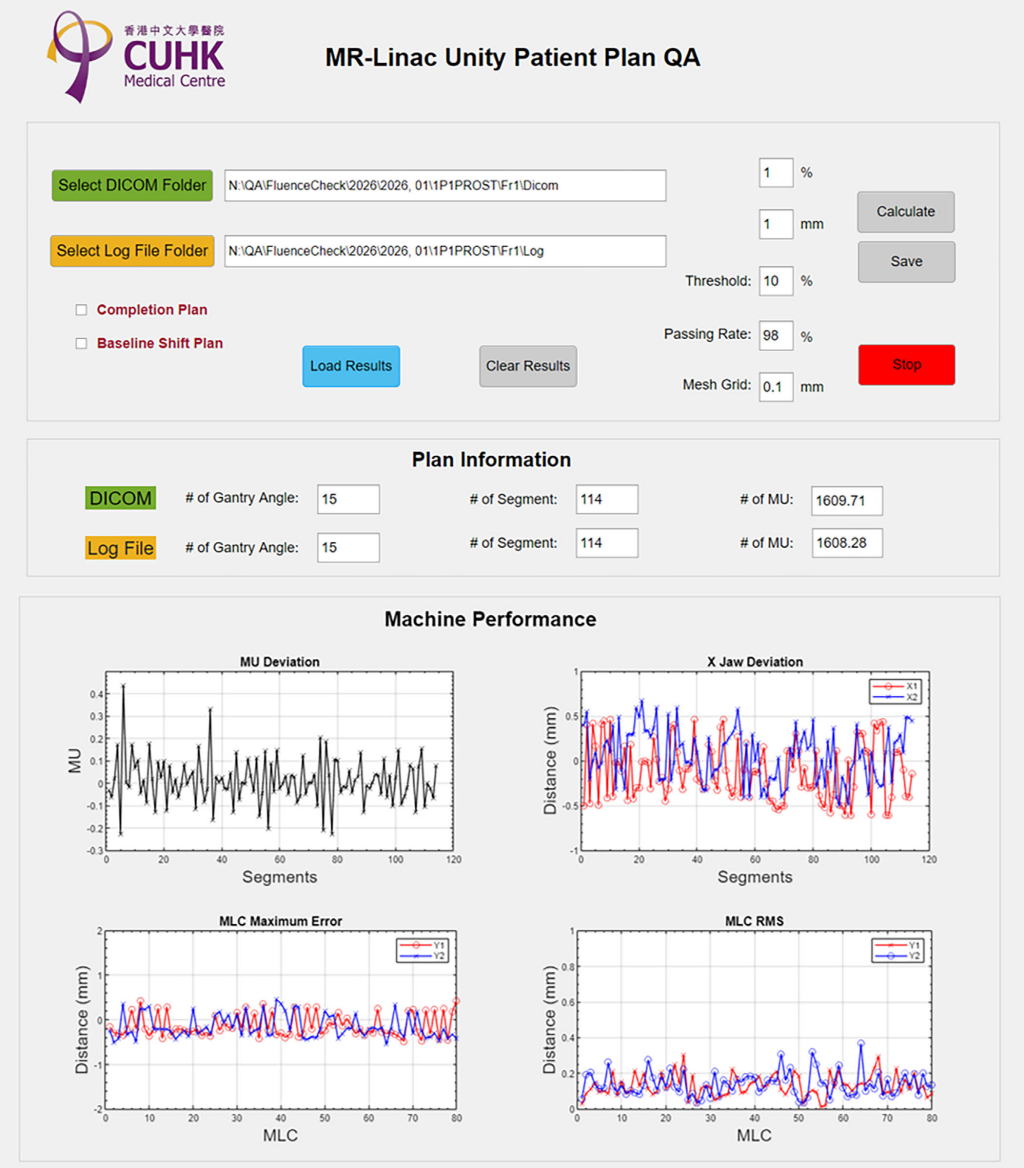
The UI for the PSQA program.

A User Interface (UI) was developed in MATLAB (MathWorks, Natick, Massachusetts, USA). The treatment plan in DICOM format and its corresponding log files were loaded from two separately designated folders, selected via the UI as shown in Figure 3. For plans involving a baseline shift or a completion plan, the corresponding option was required to be enabled, upon which a dialog window prompted the user to confirm whether the current calculation applied to the original plan or the baseline shift/completion plan. A step‐by‐step description of the program design could be found in the Supplementary Materials.

**FIGURE 4:**
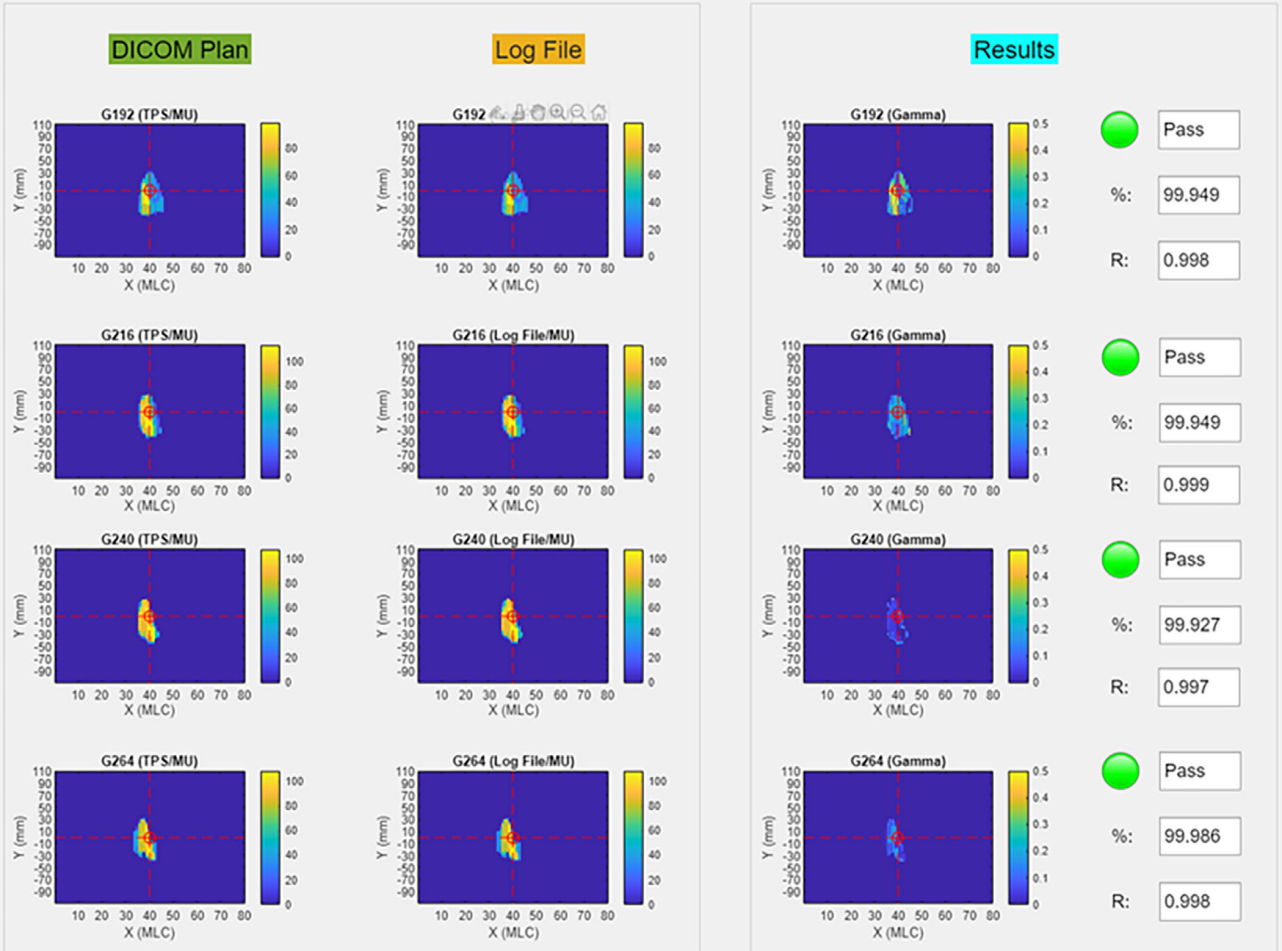
The results of the gamma analysis of fluence map generated from DICOM plan and log files.

The “Plan Information” section of the UI displayed key metadata extracted from both the DICOM plan and the log files, including the number of gantry angles, total MU, and number of segments. In the “Machine Performance” section, MU, X jaw positions, and MLC positions were compared between the DICOM plan and the log files. As shown in Figure 4, the UI presents the fluence maps generated from both the DICOM plan and the log files, along with the corresponding gamma analysis results. The system was evaluated on a total of 66 cases, comprising 3 completion plans and 2 baseline shift plans. The calculation time depended on the number of gantry angles in the plan. On a standard office PC, the calculation was completed within 10 minutes per plan.

### 2.7 Documentation

The MATLAB UI generated a structured output report containing: (1) plan metadata extracted from both the DICOM plan and log files (number of beams, total MU, segment count); (2) machine performance metrics (per‐segment MU deviation, jaw position deviation, maximum and Root Mean Square (RMS) MLC position error); and (3) gantry‐resolved gamma passing rates for each field. This report was reviewed and signed off by a qualified medical physicist prior to the patient's next treatment fraction and archived in the patient's electronic medical record, consistent with the documentation standards applied to measurement‐based QA results.

Failed PSQA cases were subsequently reviewed through measurement‐based QA with the ArcCheck (Sun Nuclear, Melbourne, FL, USA) phantom. When log file‐based QA failed but conventional measurement‐based QA passed, the discrepancy was reviewed by a qualified medical physicist to identify its root cause. The current 1% MU tolerance applied to individual segments was particularly sensitive to small MU deviations in segments resumed following a beam interruption. The documentation procedure followed the same standards described above. If the failure pattern was unexplained, recurrent, or associated with abnormal MLC or MU deviations, further investigation of machine performance, including targeted MLC QA, was warranted before proceeding with the next fraction.

## 3 RESULTS

Of the 66 plans evaluated, 12 were non‐CMM brain cases, 38 were delivered in non‐respiratory CMM mode, 11 in free‐breathing (average position) mode, 1 in free‐breathing (exhale) mode, and 4 in breath‐hold mode. 64 plans achieved passing gamma results across all fields, including all baseline shift and completion plans. Three cases (two prostate and one liver) required a completion plan. The original plans were interrupted at 35/247, 123/139, and 165/196 segments, corresponding to 3/17, 14/15, and 13/15 delivered gantry angles, respectively. The pooled gamma passing rates for the partially delivered original plans were 99.86 ± 0.47%, and for the three completion plans were 99.93 ± 0.06%, respectively. Two prostate cases required a baseline shift plan. The original plans were interrupted at 58/168 and 197/199 segments, corresponding to 5/15 and 17/17 delivered gantry angles, respectively. The pooled gamma passing rates for the partially delivered original plans were 99.93 ± 0.05%, and for the two baseline shift plans were 99.95 ± 0.05%, respectively. Eight cases experienced unexpected but resumable beam interruptions, and the pooled gamma passing rates across all fields were 99.90 ± 0.13%. Twelve non‐CMM brain cases were also evaluated using the same program and were included for completeness to benchmark performance under standard non‐gated delivery conditions.

**FIGURE 5:**
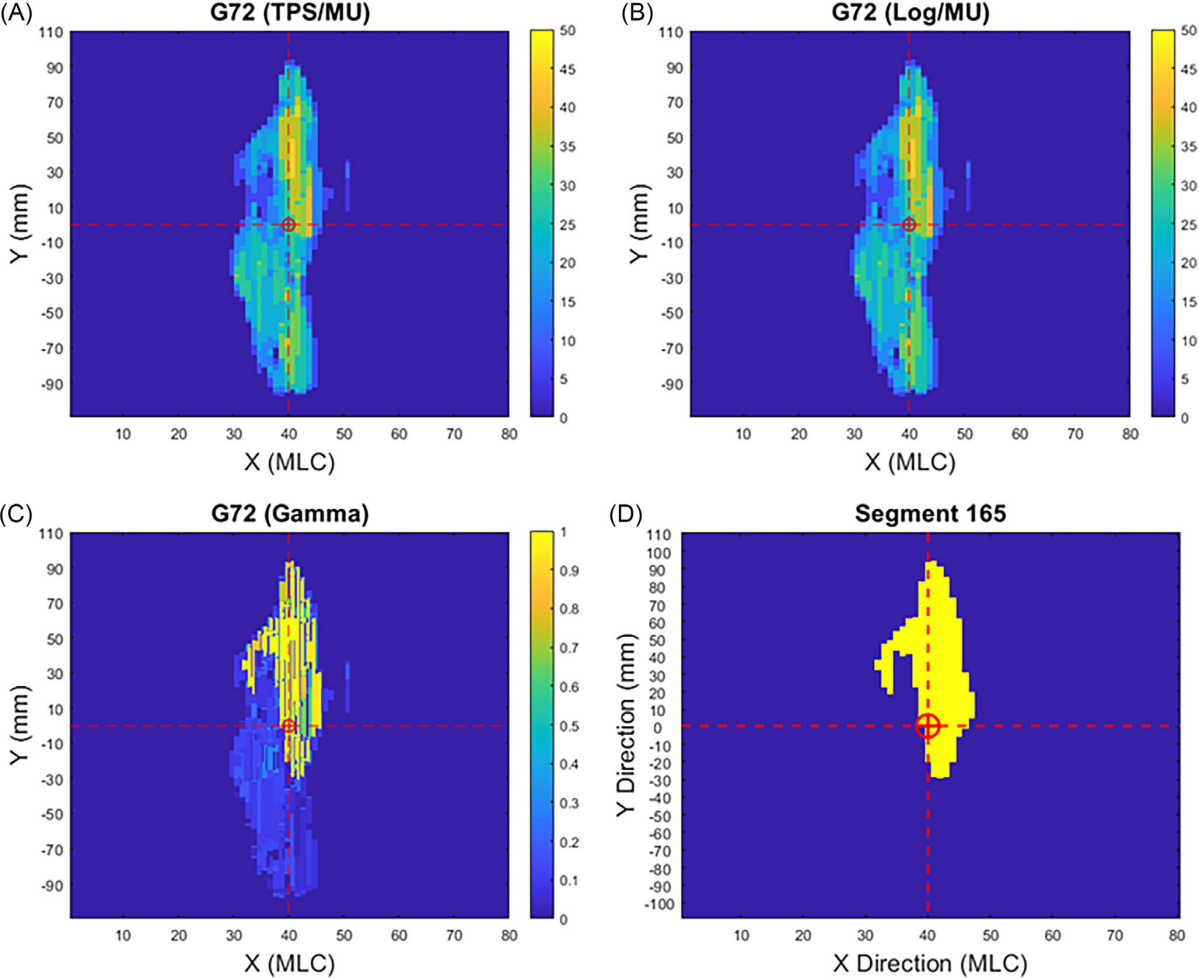
Characteristics of the failed field at gantry angle 72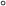. (a) Fluence map generated from the TPS plan. (b) Fluence map generated from the log file. (c) Gamma analysis results for the field. (d) Segment showing an MU deviation of 0.37.

Two plans, both corresponding to prostate treatments, contained one field each that failed the predefined gamma acceptance criteria. For these two plans, a slight difference in MU was observed between the log file and the TPS for a specific segment. Both failed cases involved a single segment with a relatively large MU deviation. In one case, a field at gantry angle 72° yielded a gamma passing rate of 86.78%, with segment No. 165 showing MU deviation of 0.37 MU between the log file and the TPS plan. In the other case, a field at gantry angle 120° yielded a gamma passing rate of 78.36%, with segment No. 61 showing MU deviation of 0.33 MU (4.2% of the planned segment MU). No significant deviation in MLC or jaw positions was observed in either case. The results of the first case are shown in Figure S1, and no significant deviation in leaf or jaw positions was observed. However, Figure S1(A) shows that, for the beam at a gantry angle of 72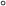, segment No. 165 had MU deviation of 0.37 MU (5.2% of the planned segment MU). between the log file and the TPS plan. As shown in Figure 5(c) and (d), the region that failed the gamma analysis overlapped with the specific segment exhibiting the 0.37 MU deviation. These cases were subsequently checked using measurement‐based QA with the ArcCheck (Sun Nuclear, Melbourne, Florida, USA) phantom. Both cases passed gamma analysis with a passing rate greater than 95% using the routine measurement‐based PSQA criteria of 3% dose difference, 2 mm distance‐to‐agreement, and a 10% threshold to fluence map. The number of beams, segments, AFS, and MU, together with the pooled average gamma passing rates and gating efficiencies, are summarized in Table 1. As CMM was not employed for brain treatments, gating efficiency was not applicable for this subgroup. Gating efficiency varied substantially across anatomical sites. Despite this variation, gamma passing rates remained consistently high across all regions.

**TABLE 1:** Plan characteristics and PSQA results.

Region	Number of beams	Number of AFS	Number of segments	Number of MU	Gamma Passing Rate (%)	Gating Efficiency (%)
**Pelvis (28)**	15.64 ± 2.67	1 (15)	149.79 ± 59.34	2232.40 ± 759.87	99.92 ± 0.29	94.17 ± 13.08
		2 (13)				
**Abdomen (14)**	15.00 ± 1.92	1 (8)	131.93 ± 76.49	2633.10 ± 1324.2	99.94 ± 0.06	78.97 ± 18.03
		2 (5)				
		3 (1)				
**Thorax (2)**	14.00 ± 2.83	1 (2)	90.50 ± 24.75	1595.80 ± 586.85	99.98 ± 0.03	37.07 ± 17.92
**Spine (10)**	16.40 ± 1.35	2 (8)	220.20 ± 54.27	3214.20 ± 620.48	99.86 ± 0.1	95.30 ± 12.15
		3 (2)				
**Brain (12)**	13.17 ± 2.72	1 (9)	84.67 ± 62.1	3573.20 ± 2179.7	99.90 ± 0.15	N/A
		2 (3)				
**All (66)**	15.12 ± 2.54	1 (34)	143.03 ± 73.34	2690.70 ± 1329.4	99.91 ± 0.21	88.33 ± 18.64
		2 (29)				
		3 (3)				

## 4 DISCUSSION

In the current study, a log file‐based PSQA program was developed and evaluated on an MR‐Linac with gating functionality. The program demonstrated robustness and sensitivity in detecting potential inconsistencies between the delivered and planned treatment parameters.

Log file‐based PSQA substantially reduces overall QA time by eliminating the need for phantom setup. By utilizing real delivery data, including MU, MLC positions, and jaw settings, this approach also minimizes additional uncertainties inherent to repeated phantom irradiations. At our institution, phantom‐based measurement using ArcCheck for the reference plan is reserved for single‐session Stereotactic Radiosurgery (SRS) cases only. For all the reference plans and online‐adapted plans, we perform independent point‐dose verification using RadCalc (Lifeline Software, Tyler, Texas, USA) as a quick dose check. In the current PSQA program, fluence map comparison was employed to evaluate agreement between the planned and log file‐derived delivery. Photon fluence serves as an effective primary indicator for this comparison in MR‐guided radiotherapy QA,^7^, ^10^ bypassing the need for complex dose calculations that require accurate magnetic field modelling for post‐treatment verification.^11^, ^12^, ^13^ Gamma analysis provides a stringent and sensitive measure for detecting anomalies in MLC positions and MU delivery. Two cases had one field that failed PSQA. Both plans were delivered with a conventional fractionation schedule of 20 fractions, whereas the majority of other cases were hypofractionated Stereotactic Body Radiation Therapy (SBRT) treatments with higher MU per segment. At low MU per segment, minor dose rate fluctuations during beam initiation can produce small discrepancies between planned and delivered MU values. Nevertheless, both cases passed measurement‐based QA with ArcCheck, achieving gamma passing rates exceeding 95% under the standard 3%/2 mm criterion.

Following the upgrade from MM to CMM, the clinical workflow benefits from several advanced features, including baseline shift correction, automatic beam gating triggered by low image quality factors, and enforcement of ATC criteria. Nevertheless, the frequent beam gating inherent to CMM presents a fundamental challenge for post‐treatment measurement‐based QA using phantoms such as ArcCheck. As the plan delivered to the phantom is a continuous, ungated plan exported directly from the TPS, and therefore does not replicate the gated delivery conditions experienced during actual treatment.^14^ Gating subdivides each planned segment into multiple sub‐segments, making delivery discontinuous and increasing the number of beam start and stop events. As a result, cumulative dosimetric uncertainty is greater compared to ungated delivery.^15^, ^16^, ^17^ Conventional measurement‐based PSQA may not adequately capture delivery imperfections arising from gating events. Log file analysis, which records the actual machine state at every delivery instant, therefore offers a more representative and clinically meaningful approach for assessing true delivery accuracy. In addition, MR‐Linac treatments are inherently more time‐consuming than those delivered on conventional linacs, and the addition of gating extends treatment duration further. Contributing factors include MR image acquisition, online re‐contouring, daily adaptive plan optimization for every fraction, the current limitation to step‐and‐shoot IMRT delivery, and a lower dose rate compared to conventional linacs. Log file‐based PSQA therefore offers a practical way to reduce machine occupancy otherwise required by measurement‐based QA.

Several limitations warrant mention. The log file may not perfectly represent the actual delivery, as discrepancies can exist between the recorded log file parameters and the true machine parameters. To mitigate this limitation, regular MLC quality assurance is recommended. As specified by AAPM TG‐142 and TG‐198, MLC leaf position accuracy should be verified qualitatively on a weekly basis and quantitatively at four cardinal gantry angles on a monthly basis.^16^, ^18^ In our institution, EPID‐based MLC position accuracy testing is performed weekly at a single gantry angle and monthly at four cardinal gantry angles, consistent with these recommendations. However, given the limited EPID field coverage of the Unity, which cannot encompass the full MLC leaf bank,^19^ a supplementary monthly film‐based picket fence test is recommended to verify leaf positioning accuracy across the complete range of leaf motion.^20^ Additionally, comprehensive calibration of the beam‐limiting devices, including the Rubicon optical leaf tracking system, is performed during scheduled preventive maintenance at 12‐month intervals. Lastly, fluence map‐based analysis does not account for patient geometry or tissue heterogeneity, which may limit its ability to reflect the true dosimetric impact on the patient.

## 5 CONCLUSION

The proposed log file‐based PSQA framework demonstrates accuracy and clinical practicality for CMM‐mode MR‐Linac treatments. It may serve as a reliable post‐treatment verification tool and reduce dependence on conventional phantom‐based QA, though further validation with larger BLS and completion plan cohorts is warranted.

## AUTHOR CONTRIBUTION STATEMENT

KY performed the experimental investigation, data analysis, and manuscript preparation. MC contributed to the experimental work and manuscript preparation. LL conceptualized the study and provided supervision. All authors reviewed and approved the final version.

## CONFLICT OF INTEREST STATEMENT

The authors declare that there are no conflicts of interest regarding the publication of this paper.

## FUNDING STATEMENT

No external funding was received for this study.

## ETHICAL STATEMENT

A written patient consent form was waived due to the retrospective nature. This study was approved by the Clinical Research Ethics Committee of CUHK Medical Centre (CREC‐202505).

## Supporting information




**Supporting Information**: acm270773‐sup‐0001‐SuppMat.docx

## Data Availability

The data that support the findings of this study are available from the corresponding author upon reasonable request.
