# Supplementary material for: Log file‐based patient‐specific quality assurance for MR‐guided radiation therapy with comprehensive motion management
Source: J Appl Clin Med Phys. 2026 Aug 25;27(9):e70773. doi: 10.1002/acm2.70773 (PMC13504375; doi:10.1002/acm2.70773)
Supplement: Supplementary file 1 [file ACM2-27-e70773-s001.docx]

Figure S1. Comparison of plan characteristics between the log file and the TPS plan for one case in which a field failed PSQA. (A) MU deviation between the log file and the TPS plan. (B) Jaw position deviation between the log file and the TPS plan. (C) Maximum MLC position error between the log file and the TPS plan. (D) Root Mean Square (RMS) of MLC position deviations across all segments.

**Step-by-Step Processing Pipeline**

1. Organize the data

- Place the TPS plan DICOM file in one folder. For completion or baseline-shift (BLS) plans, place the corresponding DICOM files in separate folders.
- Place all log files in one folder. For completion or BLS plans, place the corresponding log files in separate folders.

2. Parse the planned and delivered data

- Parse the DICOM RT plan to obtain the planned per-segment MLC positions, jaw positions, and monitor units (MU).
- Parse all log files (in CSV format) to obtain the actually delivered per-segment MLC positions, jaw positions, and MU. The software scans the Linac State column to identify contiguous blocks where the beam was actively on ("Radiation On"), with each block treated as one delivered segment.

3. Checkpoint step: merge split sub-segments in the parsed log files

- Because a single delivered segment can sometimes be split into two consecutive sub-segments, the software checks each pair of consecutive blocks before finalizing segments. It compares the leaf positions at the end of the earlier block to the leaf positions at the start of the next block. If the difference for every leaf on both banks (Y1 and Y2) falls below a small tolerance, the two blocks are judged to be the same physical segment and are merged into one, with the delivered MU from the second block added onto the first. This check is applied working backward through all detected segments, so consecutive split sub-segments can be collapsed into a single true segment.

4. Adjust for partial-delivery plan types (optional, user-selected)

- If "Completion Plan" is checked, the software trims the planned data so it only covers the segments that were actually delivered and logged.
- If "Baseline Shift Plan" is checked, the user chooses whether to check the portion of treatment given before the shift ("Original Plan," which removes the later segments) or after the shift ("BLS Plan," which removes the earlier segments), and the software trims the planned data so it only covers the segments that were actually delivered and logged.

5. Validate data alignment

- Before analysis, confirm that the number of logged segments matches the number of planned control points. If they don't match, the run stops and an alert is shown.

6. Machine performance comparison (independent of fluence-based gamma analysis)

- Compute the MU deviation (planned minus delivered) per segment.
- Compute the jaw position deviation (X1/X2) per segment.
- Compute the per-leaf MLC deviation (Y1/Y2 banks), reporting both the maximum absolute error and the RMS error across all segments for each leaf.

7. Reconstruct the delivered fluence map per beam

- For each beam, gather the logged jaw positions, the logged MLC positions, and the MU delivered per segment.
- Use these to build a 2D map showing where and how much dose was actually delivered, based on the chosen grid resolution.

8. Reconstruct the planned fluence map per beam

- For each beam, gather the planned jaw positions, the planned MLC positions, and the planned MU per segment.
- Use these to build a matching 2D map showing where and how much dose was intended to be delivered, based on the same grid resolution.

9. Gamma analysis per beam

- Perform gamma analysis using the user-specified dose-difference percentage and distance-to-agreement (DTA), with global normalization referenced to the maximum pixel value in the cropped planned (TPS) map.
- Compute the gamma passing rate as the percentage of pixels (restricted to those with planned dose above a user-defined threshold percentage of the beam's maximum dose) that have a gamma value below 1.

10. Pass/fail determination and display

- For each beam, mark it "Pass" (green lamp) if the passing rate exceeds the defined threshold, or "Fail" (red lamp) otherwise. Results are displayed immediately for each beam.

11. Save and reload results

- The "Save" button stores all fluence maps, gamma maps, machine performance data, and analysis settings (DTA, dose difference criterion, fluence threshold, resolution, pass-rate criterion) to a .mat file.
- The "Load Results" button reconstructs the full display from a saved file without needing to recompute anything.
